# Beneficial Effects of *Holothuria leucospilota* Polysaccharides on Fermentability In Vivo and In Vitro

**DOI:** 10.3390/foods10081884

**Published:** 2021-08-15

**Authors:** Wanting Wang, Yiqiong Yuan, Jun Cao, Xuanri Shen, Chuan Li

**Affiliations:** 1 Engineering Research Center of Utilization of Tropical Polysaccharide Resources of Ministry of Education, Hainan University, Haikou 570228, China; wangwanting97@163.com (W.W.); yiqiongyuan@126.com (Y.Y.); juncaoyd2007@126.com (J.C.); shenxuanri2009@163.com (X.S.); 2 Hainan Provincial Engineering Research Centre of Aquatic Resources Efficient Utilization in the South China Sea, Hainan University, Haikou 570228, China; 3 School of Food Science and Engineering, Hainan University, Haikou 570228, China; 4 Collaborative Innovation Center of Provincial and Ministerial Co-Construction for Marine Food Deep Processing, Dalian Polytechnic University, Dalian 116034, China

**Keywords:** *Holothuria leucospilota*, polysaccharides, fermentation behaviors, antioxidant activity

## Abstract

This work aimed to investigate the in-vitro and in-vivo fermentation behaviors of *Holothuria leucospilota Polysaccharides* (HLP) and the impact on mouse liver antioxidant activity. HLP showed excellent fermentability during in vitro experiments, which was characterized by increased levels of total sugar consumption and short-chain fatty acids (SCFAs). During in vitro fecal fermentation, the fucose contents in the HLP fermentation products (0.174 mg/mL) were higher than those of xylose and galactosamine during the first three hours, and fucose disappeared after 24 h. The concentrations of the generated SCFAs increased to 111.13 mmol/mL after in-vitro fermentation at 48 h. After 28 days of oral administration, the SCFA contents that were detected in the feces of mice treated with high HLP doses were significantly higher than those in the feces of mice treated with lower doses and the normal group. In addition, histological observations demonstrated that HLP increased the number of goblet cells without causing hepatocellular injury. Moreover, the increased glutathione peroxidase (GSH-Px) and superoxidase dismutase (SOD) activities and decreased malondialdehyde (MDA) contents in the mouse livers treated with HLP suggested the good performance of HLP with respect to liver antioxidants.

## 1. Introduction

Polysaccharides are inextricably associated with human health and exist in our foods, which are polymeric carbohydrate macromolecules consisting of multiple monosaccharide units linked by glycosides [1,2]. Recently, developing more bio-based or green chemical food ingredients has become a global trend to meet the increasingly diversified and healthy diet [3]. Exploring new polysaccharide material sources with useable characteristics has attracted the interest of researchers, as polysaccharides with different functions have been isolated from various plants and animals, bacteria, and fungi [4,5]. Despite the lack of large-scale industrial applications, it still makes great sense to understand the available natural resources worldwide [6]. Nondigestible polysaccharides cannot be absorbed immediately by the gastrointestinal digestive system [7]. Trillions of microorganisms coexist in the human intestine and are collectively referred to as the gut microbiota and play a vital role in human diets and host health [8]. However, indigestible polysaccharides can pass through the gastrointestinal tract to the distal gut and then be fermented by the gut microbiota [9] and produce some beneficial metabolites, such as short-chain fatty acids (SCFAs) [10]. SCFAs greatly influence the host physiology and energy homeostasis, especially in maintaining epithelial barrier function and preventing colorectal cancer [11].

As an edible marine resource and traditional medicine, sea cucumber has been used in many Asian countries to treat diverse diseases based on its antioxidant, antithrombotic, and antitumor activities. *Holothuria leucospilota* (Echinodermata: Holothuroidea, *H. leucospilota*) is a tropical sea cucumber species present in the Indo-Pacific region [12]. Among the varieties of active substances in its body wall, polysaccharides are the most noticeable ingredient, accounting for up to 31% of the total organic matter of dried sea cucumbers [13]. The HLP has been shown to be made of typical acidic polysaccharides with a backbone of intercalated 1,4-GlcA and 1,3-GalNAc, branched with fucose with different sulfate patterns and is composed of galactosamine, fucose, and glucuronic acids with ratios of 39.08%, 35.72%, and 10.72%, respectively [14,15]. Our previous works reported that HLP alleviated the symptoms of type 2 diabetes mellitus in rats by improving the gut microbiome [16]. In addition, Yuan et al. showed its excellent antioxidant activity after gastric and intestinal digestion in vitro [17]. However, the information regarding how HLP affects host health is still insufficient. The in-vitro and in-vivo fermentation characteristics and antioxidant activities of HLP have not been comparably explored.

Accordingly, the present study was designed to investigate the in-vitro and in-vivo fermentation behaviors of HLP by characterizing the pH, total sugar, reducing sugar, free monosaccharide, and SCFAs. In addition, the liver malondialdehyde (MDA), glutathione peroxidase (GSH-Px), and superoxidase dismutase (SOD) activities in mice were determined after 28 days of oral administration. This study is expected to promote the application of functional HLP in the food and pharmacological industries.

## 2. Materials and Methods

### 2.1. Preparation of the Polysaccharides

The dried sea cucumbers, *H. leucospilota* (80–130 mm, 22–47 g), were purchased from the Market Property Development Co., Ltd. (Haikou, China). Professor Yongqin Feng of Hainan University confirmed the species of the sea cucumber sample.

The polysaccharides were extracted from the *H. leucospilota* sample according to a previously reported method with slight modifications [15]. The dried sea cucumber body wall powder was mixed with sodium acetate buffer, papain, EDTA solution, and cysteine. The mixture was centrifuged (4500× *g*, 15 min, 4 °C), and a chlorohexadecyl pyridinium (CPC) solution was added to the clear supernatant. After 12 h, the mixture was centrifuged (4500× *g*, 15 min, 4 °C), and the precipitated polysaccharides were redissolved in NaCl: ethanol (100:15, *v*/*v*) solution with 95% ethanol. The sediments were collected after centrifugation (3500× *g*, 15 min, 4 °C) and were reconstituted with distilled water. After decolorization and deproteinization, the polysaccharides were dialyzed with distilled water for 72 h. The HLP with extremely low protein content (0.26%) was obtained by concentration and lyophilization.

### 2.2. In-Vitro Fermentation of HLP

#### 2.2.1. Preparation of Human Intestinal Microbiota

Fecal inocula were collected from eight healthy volunteers who had not taken any antibiotics within three months (male: female = 1:1, average age 20–30 years). Equal amounts of feces from each person were pooled. Samples of 50 g were diluted with 200 mL of Dulbecco’s phosphate-buffered saline (10%, D-PBS), which was followed by homogenization. The mixture was filtered through four-layer nylon gauze, and the obtained filtrate was collected.

#### 2.2.2. In-Vitro Fermentation Procedure

The polysaccharides solution, fermentation medium solution, and fecal solution were added to each ampoule tube. The sealed ampoule tubes were incubated in a shaker (250 rpm, 37 °C) for 6, 12, 24, and 48 h. After incubation, the anaerobic tubes were placed in an ice water bath for 10 min for termination. After centrifugation (8000× *g*, 10 min), the supernatant was transferred and stored at −20 °C before analysis.

#### 2.2.3. Determination of Total Sugar, Reducing Sugar, and Free Monosaccharides in the Fermented Products

The total sugar contents were determined by the phenol sulfuric acid method with glucose as the standard. The dinitrosalicylic acid (DNS) method was utilized to determine the reducing sugar contents [18]. The free monosaccharides were determined by gas chromatography (GC) according to the methods of a previous study [19].

#### 2.2.4. Determination of pH and SCFA Content

The fermentation supernatant pH levels were measured with an MP511 Lab pH Meter (Sanxin Apparatus, Shanghai, China).

Anhydrous ethanol and the fermentation supernatants (or SCFAs standard solutions) were added to tubes and mixed. The mixtures were supplemented with concentrated sulfuric acid and n-hexane. The upper organic phase was used to determine the concentrations of SCFAs. SCFAs analysis was performed by an Agilent 6890A GC equipped with a flame ionization detector (FID) and HP-FFAP column (30 m × 0.32 mm × 0.25 μm, Agilent Technologies, Santa Clara, CA, USA).

### 2.3. In Vivo Fermentation of HLP

Thirty-two male Kunming mice were purchased from the Tianqin Biotechnology Co., Ltd. (Changsha, China), Certificate Number: SCXK (Xiang) 2014−0011. The mice were housed under controlled environmental conditions at a temperature of 23 ± 1 °C and a 12/12 h light/dark cycle with ad libitum water and food. All procedures were performed according to the Guidelines of the Care and Use of Laboratory Animals. The experiments were approved by the Animal Experimentation Ethics Committee of Hainan Medical University.

The mice were randomly divided into four groups and received intragastric administrations once every day: low-dose (HLP-L, 50 mg/kg body weight) group; medium-dose (HLP-M, 100 mg/kg body weight) group; high-dose (HLP-H, 200 mg/kg body weight) group; and an equal volume of distilled water as the normal group (ND). The health of the mice was recorded daily for 28 days. Fecal samples from each group were collected after gavage every fortnight. Finally, the mice were sacrificed by cervical dislocation after anesthesia with chloral hydrate. The liver and small intestine tissues were taken under sterile conditions and transferred to a −80 °C refrigerator for preservation.

### 2.4. Liver Antioxidant Activity Assay

The mouse liver tissues were added to ice-cold physiological saline, homogenized, and centrifuged (4 °C, 3000× *g* and 10 min). The malondialdehyde (MDA) contents and superoxide dismutase (SOD) and glutathione peroxidase (GSH-Px) activities in the liver were measured with the corresponding kits (Jiancheng, Nanjing, China).

### 2.5. Histological Examinations

The ileum and liver samples were fixed in 10% neutral buffered formalin solution for histopathological processing. After dehydration, the fixed tissues were embedded in paraffin and were sectioned and dyed with hematoxylin-eosin (H&E). The stained areas were viewed with an optical microscope (Nikon Eclipse, Tokyo, Japan).

### 2.6. Statistical Analysis

The results were presented as the means ± standard deviation (SD). The data were analyzed using one-way analysis of variance (ANOVA) using SPSS 25.0 software (SPSS Inc., Chicago, IL, USA) and compared using Duncan’s test. *p* < 0.05 was considered to be significantly different.

## 3. Results and Discussion

### 3.1. Changes in Total Sugar, Reducing Sugar, and Free Monosaccharide Contents during In-Vitro Fermentation

The enzymes that are encoded by the human microbiota cleave the glycosidic bonds of carbohydrates to fermentable saccharides [20]. The breakdown of glycosidic bonds leads to the release of some terminal, reducing sugars of the polysaccharides. Hence, the total sugar and reducing sugar contents are useful to evaluate the fermentation extent of polysaccharides [21]. As illustrated in Table 1, the initial total sugar concentration was 1.099 mg/mL. After six hours of fermentation, the total sugar concentration decreased by 56.77% to 0.475 mg/mL, and the rate of decrease was faster than that afterward. The total sugar concentration was 0.218 mg/mL at the end of fermentation, which decreased by 0.881 mg/mL from the initial concentration. However, despite being widely used in many reported studies, the phenol-sulfuric method is not specific enough to determine the residual carbohydrate contents from in-vitro fermentation. Consequently, the total sugar contents that were determined at 0 h were not consistent with the original amount of HLP in the culture medium.

Meanwhile, the concentration of reducing sugar was initially 0.432 mg/mL, and it increased to 0.833 mg/mL after three hours, which was followed by a sharp drop to 0.274 mg/mL at six hours. These changes occurred because the generation rate of reducing sugar was faster than its consumption rate at the beginning of fermentation. Thus, the total sugar concentrations showed a declining trend throughout the fermentation process, while the reducing sugar concentrations increased and then followed by a decrease. Similar changes were observed in a study of the in-vitro fermentation of polysaccharides from *Sargassum thunbergii* [21]. The free monosaccharide contents in the fermented products reflect the hierarchy of polysaccharide utilization [22]. As shown in Table 1, free monosaccharides were detected after three hours of fermentation (the fucose, xylose, and galactosamine concentrations were 0.174, 0.063, and 0.017 mg/mL, respectively). The results showed that HLP was degraded into free monosaccharides by the gut microbiota combined with reducing sugar variations. The degradation rate was lower than the monosaccharide utilization rate in the first three hours. Furthermore, fucose was not detected after 24 h of fermentation, while xylose and galactosamine were not detected after 48 h of fermentation. The disappearance of free monosaccharides occurred because the gut microbiota rapidly utilized them. This result was in accordance with the changing trend reported in another study of *Oudemansiella radicata* polysaccharides [19]. Interestingly, the fucose concentrations were higher than those of xylose and galactosamine from 3 h to 12 h, and fucose disappeared earlier, which was different from xylose and galactosamine. It has been reported that the constituent monosaccharides and spatial structures significantly influence the fermentability of polysaccharides [23]. Fucose was consumed on the branches with a residual content of xylose, and then, galactosamine in the backbone was consumed [14]. In addition, diverse fecal bacteria prefer to utilize different monosaccharides that are liberated from polysaccharides [24]. Thus, it was speculated that fucose might be linked to the side chain in the HLP structure and be more prone to be utilized or degraded by the microbiota [14,21].

### 3.2. Changes in pH Values and SCFA Contents during In-Vitro Fermentation

The variations in pH values of the fermented products reflected the fermentation surroundings and are summarized in Table 2. The pH value in the human colon is approximately 5–7, and this slight acidity is due to some acidic metabolites (such as SCFAs) that are produced by intestinal microorganisms. The initial pH value of the HLP fermentation system exhibited slight acidity at 6.87 (0 h). Thereafter, a rapid and significant pH decrease (to 4.49) was observed during HLP fermentation from 0 h to 12 h (*p* < 0.05), which indicated that some acid metabolites were produced during fermentation. Many other studies have also reported that the pH values of fermentation solutions declined with the extension of fermentation time [25]. However, the pH values of the fermented products exhibited an increasing trend after 24 h, which were similar to the results reported by Gao et al. [26]. Presumably, this might be due to the production of some alkaline metabolites in the culture medium. A weakly acidic environment has been proven to promote the proliferation of beneficial bacteria in the intestinal tract and inhibit the growth of harmful bacterial [27]. Thus, HLP promotes gut health by maintaining acidic environments.

SCFAs, such as acetic acid, propionic acid, and butyric acid, which are produced during fermentation, play an indispensable role in sustaining human health [28]. Nevertheless, it is difficult to determine the SCFA contents that are generated in vivo, as over 95% of SCFAs are rapidly absorbed and metabolized by the host. The SCFA concentrations in fecal samples are greatly affected by the intestinal transport times and cannot accurately reflect the proximal colon environment [29]. Hence, combining in-vitro and in-vivo fermentation methods more accurately describes the actual fermentation behavior of polysaccharides. The total SCFAs concentration increased with fermentation time (Table 2). After 48 h of fermentation, the total SCFAs content increased by 111.13 mmol/L. The SCFA amounts are related to the microbial compositions, substrate sources, and intestinal action times [30]. Therefore, the variety of SCFAs and their concentrations during fermentation are generally used to determine polysaccharide fermentability.

Acetic acid is usually converted from pyruvate and is utilized by the liver, muscles, and peripheral tissues through blood circulation and is the most abundant SCFA in the colon [31]. As histone deacetylase inhibitors, propionic and butyric acids participate in the immune regulation process [32]. Among the various SCFAs, the acetic acid, propionic acid, and butyric acid contents were comparably higher and changed more distinctly. The acetic acid concentrations showed a continuously increasing trend from the initial 12 h period and decreased gradually from 12 to 24 h. Nevertheless, the propionic acid and butyric acid contents increased gradually throughout the entire process. This result was probably explained by the acetate CoA-transferase route, namely the gut bacteria further metabolized acetate to butyrate via butyryl-CoA [33]. It is noteworthy that the acetic acid concentration reached its peak value at 12 h, which was consistent with the trend of pH values. The fact that the pH of acetic acid is lower than that of other SCFAs at the same concentrations contributes to this phenomenon. Additionally, the butyric acid concentration was evidently high because the weakly acidic environment was conducive to the metabolism and reproduction of butyrate-producing bacteria. The growth of pH-sensitive pathogens and certain carcinogenic saprophytes (such as *Clostridium* and *Enterobacteriaceae*) was effectively inhibited [29]. In addition, Chen et al. also found that the isobutyric acid and isovaleric acid levels were relatively lower, which resulted from few intestinal bacteria producing isobutyrate and isovalerate [4,19]. HLP may be degraded and utilized by the gut microbiota during in-vitro fermentation, which produced abundant SCFAs.

### 3.3. Changes in pH Values and SCFA Contents in Mouse Feces

**Figure 1:**
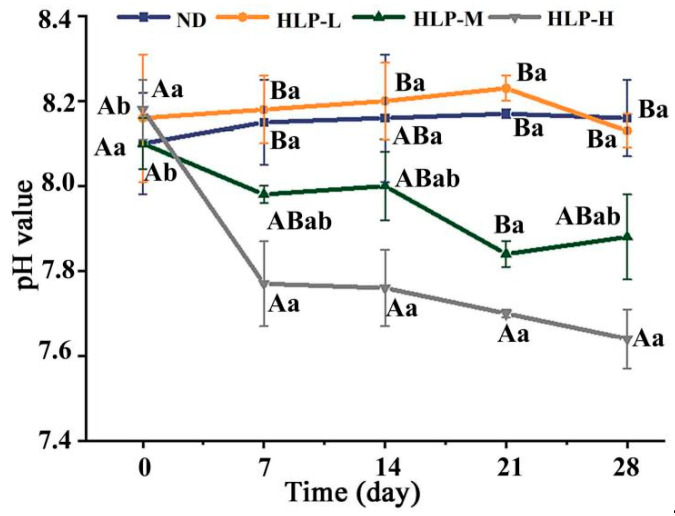
Changes in pH values in the mouse feces among different groups. The different uppercase and lowercase letters around the points represent significant differences in various groups and times (day), respectively (*p* < 0.05).

The intestinal microenvironment is a complex ecological and reaction system. The appropriately decreased pH values in the intestine contributed to the growth of probiotics and inhibition of pathogens [34]. The fecal pH value is generally used as an indicator to respond to intestinal health. Based on seven days of oral administration, significant differences in pH values appeared among the different groups (Figure 1). In particular, the fecal pH values of the HLP-H and HLP-M groups were obviously lower than those of the HLP-L and ND groups. This result suggested that the intake of HLP decreased pH in the mouse intestine and that a high-dose intake had a greater influence on pH. The colon and fecal SCFA contents are closely associated with human health.

**Figure 2:**
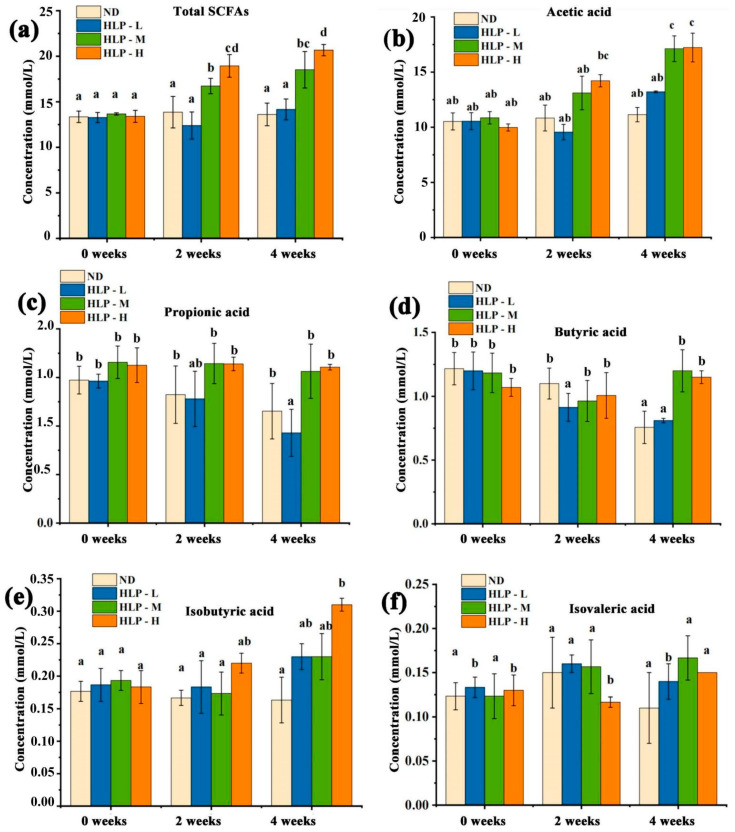
Changes in total SCFAs (**a**), acetic acid (**b**), propionic acid (**c**), butyric acid (**d**), isobutyric acid (**e**), and isovaleric acid (**f**) contents in mouse feces. Values with different superscripts represent significant differences (*p* < 0.05).

When compared to the ND group, after HLP treatments for two and four weeks, the total SCFAs concentrations in the HLP-M and HLP-H groups increased significantly (*p* < 0.05, Figure 2). The HLP-H group increased most notably among all groups after four weeks, from 13.4 mmol/L to 20.67 mmol/L. The total SCFAs contents after four weeks were significantly higher than those after two weeks. This result was consistent with the changing trend of pH values in mouse feces and indicated the close relationship between the mouse fecal pH values and SCFA contents in the mouse colon. After four weeks of continuous gavage, both the HLP-M and HLP-H doses caused significant increases in the acetic acid and butyric acid concentrations in feces compared to the ND group (*p* < 0.05). In addition, the fecal isobutyric acid concentrations in the HLP-H group were significantly higher than those in the ND group after four weeks (*p* < 0.05). This result suggested that the intestinal environment of the medium-dose and high-dose HLP-treated mice was affected for four weeks.

### 3.4. Histological Analysis of the Ileum and Liver

**Figure 3:**
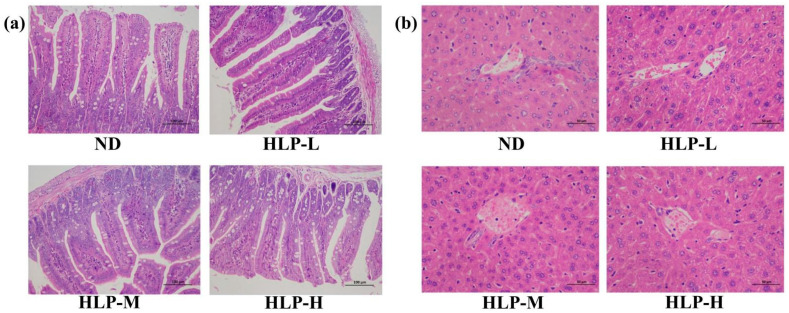
Histological examination of the ileum (**a**) and liver (**b**) by H&E staining (200×).

The ileum in mice of the HLP-treated groups exhibited normal histological features (Figure 3a). The ileal villi lengths and crypt depths of all mouse groups were similar. However, the goblet cell numbers among the villi clearly increased in the ileum compared to those of the ND group. Goblet cells are one of the most distinct cell types in the epithelium for intrinsic mucosal immunity and synthesize and secrete mucin, which forms a mucosal barrier to protect epithelial cells [35]. As shown in Figure 3b, the hepatocytes in the ND group were arranged in an orderly manner and were distributed radially around the hepatic cords. The liver tissue structures of the HLP-treated groups were normal and clearly showed the nucleolus, central veins, and abundant cytoplasm. This result implied that, from a pathological perspective, HLP administration would not cause hepatocellular injury.

### 3.5. Effects of HLP on Oxidative Stress Parameters in Mouse Livers

Oxidative stress, with excessive formation of reactive oxygen species (ROS), is a significant participant in diabetes and cardiovascular disease development and progression. High ROS levels damage cell structures and induce lipid peroxidation, which causes further injury to the surrounding tissues. Meanwhile, ROS also reduces antioxidant enzyme activities in the body, which results in further oxidative damage [36]. As important antioxidant enzymes in the defense system, both GSH-Px and SOD play crucial roles in protecting tissues against oxidative damage that is induced by superoxide anions [37]. GSH-Px catalyzes the formation of glutathione and reacts with ROS to decrease oxidant levels and lipid oxidation. SOD is the most strongly antioxidant enzyme in the first line of defense against ROS.

**Figure 4:**
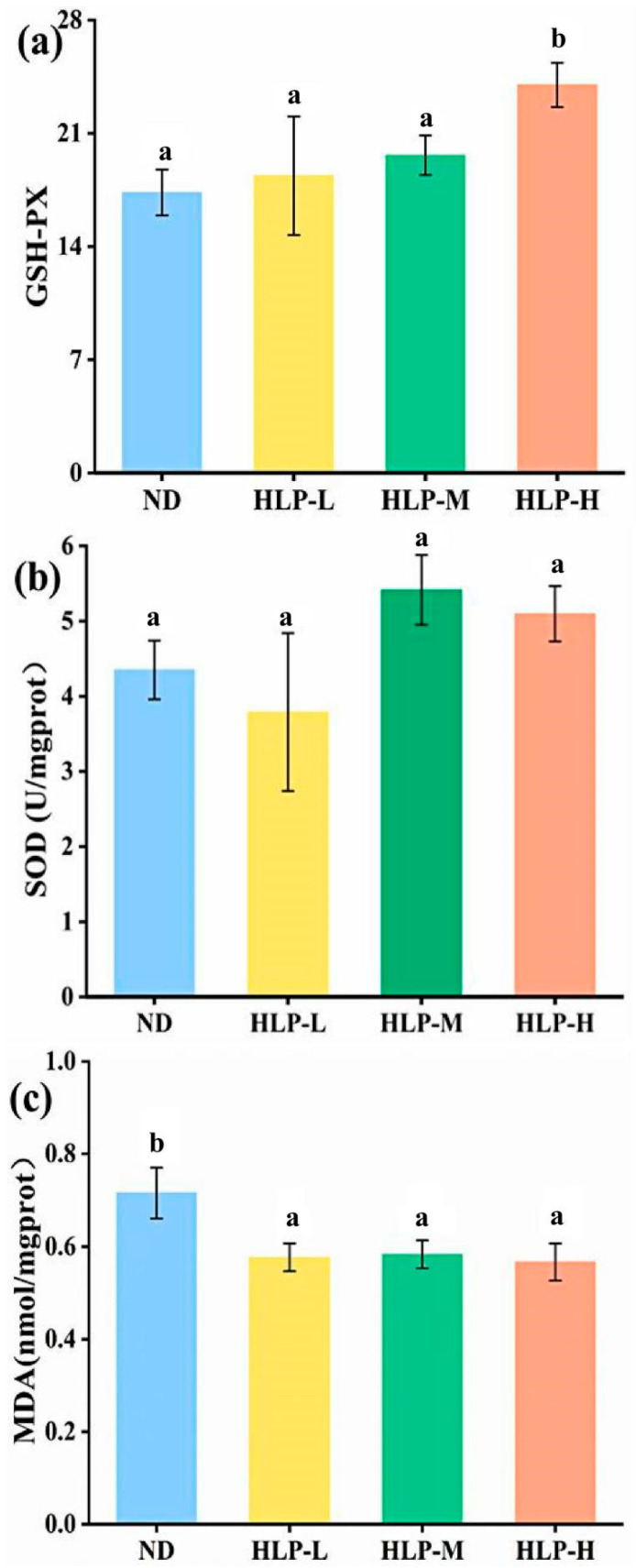
Effects of HLP on GSH-Px activity (**a**), SOD activity (**b**), and MDA content (**c**) in the mouse livers. Values with different letters are significantly different (*p* < 0.05).

The GSH-Px activities in the livers of the HLP-H group were significantly higher (*p* < 0.05) than those of the ND group (Figure 4a). Although there were no differences (*p* > 0.05) in SOD activity among the various groups, the SOD activities in the livers of the HLP-M and HLP-H groups were slightly higher than those of the ND group (Figure 4b). The increasing GSH-Px and SOD levels in the liver indicated that high-dose HLP enhanced the antioxidant defense system’s reaction to oxidative stress. MDA, a lipid peroxidation secondary product, is involved in forming lipid radicals and reflects the degree of cytotoxicity and cellular damage [38]. The liver MDA levels in the HLP-treated groups significantly declined compared with those in the ND group (Figure 4c). However, no significant differences were observed among the different HLP-treated groups in reducing the MDA levels (*p* > 0.05). This result indicated that HLP exhibited antioxidant activity by activating endogenous antioxidative enzymes.

## 4. Conclusions

The polysaccharides from *H. leucospilota* were utilized and decomposed into free monosaccharides by the intestinal microbiota. During in-vitro human fecal fermentation, the pH values and total sugar contents decreased, while various SCFA contents increased. After 28 days of high-dose treatment, the acetic and isobutyric acid levels in mouse feces increased significantly. Additionally, HLP exhibited antioxidant activity by enhancing the GSH-Px and SOD levels and decreasing the MDA contents in mouse livers. This study suggested that HLP plays an important role in potential health benefits by promoting intestinal health and antioxidant activity.

## Figures and Tables

**Table 1:** Changes in total sugar, reducing sugar and free monosaccharide contents for in-vitro fermentation.

Time (h)	Total Sugar (mg/mL)	Reducing Sugar (mg/mL)	Fucose (mg/mL)	Xylose (mg/mL)	Galactosamine (mg/mL)
0	1.099 ± 0.177 ^d^	0.432 ± 0.014 ^c^	ND	ND	ND
3	0.928 ± 0.051 ^c^	0.833 ± 0.025 ^d^	0.174 ± 0.004 ^c^	0.063 ± 0.009 ^b^	0.017 ± 0.002 ^a^
6	0.475 ± 0.066 ^b^	0.274 ± 0.034 ^b^	0.134 ± 0.001 ^b^	0.086 ± 0.004 ^c^	0.016 ± 0.001 ^a^
12	0.265 ± 0.018 ^a^	0.104 ± 0.004 ^a^	0.119 ± 0.009 ^a^	0.029 ± 0.001 ^a^	0.029 ± 0.004 ^b^
24	0.234 ± 0.019 ^a^	0.102 ± 0.004 ^a^	ND	0.036 ± 0.001 ^a^	0.016 ± 0.007 ^a^
48	0.218 ± 0.009 ^a^	0.097 ± 0.002 ^a^	ND	ND	ND

Values are the means ± SD of three replicates. Data that are followed by different letters in the same column are significantly different (*p* < 0.05).

**Table 2:** Changes in pH and SCFAs in fermented cultures that were supplemented with HLP at different times during in vitro fermentation.

Time (h)	0	6	12	24	48
pH	6.87 ± 0.01 ^e^	4.59 ± 0.02 ^f^	4.49 ± 0.00 ^a^	5.55 ± 0.01 ^c^	5.91 ± 0.02 ^d^
Acetic acid (mmol/mL)	9.65 ± 0.26 ^a^	39.00 ± 5.90 ^b,c^	70.71 ± 1.48 ^d^	34.99 ± 1.3 ^b^	45.53 ± 9.86 ^b^
Propionic acid (mmol/mL)	2.93 ± 0.05 ^a^	7.04 ± 1.01 ^b^	16.00 ± 0.92 ^c^	16.26 ± 0.92 ^c^	18.26 ± 3.93 ^c^
Isobutyric acid (mmol/mL)	0.26 ± 0.01^a^	0.36 ± 0.04 ^a^	0.15 ± 0.02 ^a^	2.32 ± 0.13 ^c^	3.15 ± 0.58 ^d^
Butyric acid (mmol/mL)	2.70 ± 0.31 ^a^	3.09 ± 0.84 ^a^	3.53 ± 0.43 ^a^	28.30 ± 1.61 ^b^	33.76 ± 7.20 ^c^
Isovaleric acid (mmol/mL)	0.40 ± 0.04 ^a^	0.63 ± 0.02 ^a^	0.32 ± 0.03 ^a^	2.99 ± 0.18 ^b^	3.93 ± 0.68 ^c^
Pentanoic acid (mmol/mL)	0.64 ± 0.08 ^a^	0.42 ± 0.02 ^a^	0.41 ± 0.07 ^a^	4.62 ± 0.18 ^b^	6.51 ± 1.34 ^c^
Total (mmol/mL)	16.58 ± 0.74 ^a^	50.54 ± 5.99 ^b^	91.12 ± 2.88 ^c^	89.47 ± 4.30 ^c^	111.13 ± 23.19 ^d^

Values are the means ± SD of three replicates. Data that are followed by different letters on the same line are significantly different (*p* < 0.05).

## Data Availability

All raw data supporting reported results is available from authors upon request.
